# STIM1 promotes angiogenesis by reducing exosomal miR-145 in breast cancer MDA-MB-231 cells

**DOI:** 10.1038/s41419-020-03304-0

**Published:** 2021-01-04

**Authors:** Shunli Pan, Xiaoxia Zhao, Chen Shao, Bingjie Fu, Yingying Huang, Ning Zhang, Xiaojing Dou, Zhe Zhang, Yuling Qiu, Ran Wang, Meihua Jin, Dexin Kong

**Affiliations:** 1 Tianjin Key Laboratory on Technologies Enabling Development of Clinical Therapeutics and Diagnostics, School of Pharmacy, Tianjin Medical University, 300070 Tianjin, China; 2 School of Medicine, Tianjin Tianshi College, Tianyuan University, 301700 Tianjin, China

**Keywords:** Tumour angiogenesis, Cancer microenvironment

## Abstract

Cancer cells secrete abundant exosomes, and the secretion can be promoted by an increase of intracellular Ca^2+^. Stromal interaction molecule 1 (STIM1) plays a key role in shaping Ca^2+^ signals. MicroRNAs (miRNAs) have been reported to be potential therapeutic targets for many diseases, including breast cancer. Recently, we investigated the effect of exosomes from STIM1-knockout breast cancer MDA-MB-231 cells (Exo-STIM1-KO), and from SKF96365-treated MDA-MB-231 cells (Exo-SKF) on angiogenesis in human umbilical vein endothelial cells (HUVECs) and nude mice. The exosomes Exo-STIM1-KO and Exo-SKF inhibited tube formation by HUVECs remarkably. The miR-145 was increased in SKF96365 treated or STIM1-knockout MDA-MB-231 cells, Exo-SKF and Exo-STIM1-KO, and HUVECs treated with Exo-SKF or Exo-STIM1-KO. Moreover, the expressions of insulin receptor substrate 1 (IRS1), which is the target of miR-145, and the downstream proteins such as Akt/mammalian target of rapamycin (mTOR), Raf/extracellular signal regulated-protein kinase (ERK), and p38 were markedly inhibited in HUVECs treated with Exo-SKF or Exo-STIM1-KO. Matrigel plug assay in vivo showed that tumor angiogenesis was suppressed in Exo-STIM1-KO, but promoted when miR-145 antagomir was added. Taken together, our findings suggest that STIM1 promotes angiogenesis by reducing exosomal miR-145 in breast cancer MDA-MB-231 cells.

## Introduction

Breast cancer is the most frequently diagnosed cancer and the leading cause of cancer death among females worldwide^1^. Among all breast cancer subtypes, 10–24% of invasive breast cancers are triple-negative showing poor prognosis^2^. Angiogenesis, defined as the formation of new blood vessels from a preexisting vascular network, has been known to play pivotal roles in tumor progression^3^. Tumor angiogenesis sustains enzymatic degradation of the vessel’s basement membrane, endothelial cell proliferation, migration, sprouting, branching, and tube formation, therefore providing oxygen and nutrition to tumor cells for proliferation and metastasis.

Exosomes are membrane-derived vesicles of endocytic origin ranging in size from 30 to 100 nm^4^. Exosomes are secreted by most cells such as epithelial cells, endothelial cells, mast cells, stem cells, T cells, B cells, dendritic cells, and cancer cells^5^. Accumulating evidence suggests that exosomes secreted from cancer cells are involved in tumor growth, tumorigenesis, angiogenesis, tumor immune escape, drug resistance, and metastasis^6^. Functional contents of exosomes such as lipids (ceramide, cholesterol, phosphatidylserine, etc.), proteins (adhesion molecules, MHC class II, tetraspanin, etc.), and nucleic acids (DNA, mRNA, miRNA, etc.), could trigger specific intracellular cascades and affect the gene expression of the recipient cells^6^. miRNAs are a type of endogenous and small noncoding RNAs (~20–25 nt) that play important roles in cancer development as oncogenes or tumor suppressors^7^.

Intracellular calcium ions (Ca^2+^) are universal second messengers that are intimately related to a number of diverse cellular processes, including cell differentiation, proliferation, and apoptosis^8^. Store-operated Ca^2+^ entry (SOCE) is the predominant Ca^2+^ entry mechanism ubiquitous in different cell types, and stromal interaction molecule 1 (STIM1) and Orai1 (also named CRACM1) are responsible for SOCE. STIM1, a transmembrane protein located in the endoplasmic reticulum (ER), activates Ca^2+^ influx through plasma membrane Ca^2+^ channels under stimuli, triggering a transient depletion of the intraluminal Ca^2+^ ^9^. Ca^2+^ store depletion leads to a rapid translocation of STIM1 into puncta that accumulate near the plasma membrane, and STIM1 operates via interaction with Orai1 and regulates the SOCE^10^.

The effect of Ca^2+^ level change on tumor angiogenesis through regulating exosome secretion has not been reported. In the present study, we used A23187 (calcium ionophore) or SKF96365 (a pharmacological store-operated Ca^2+^ influx inhibitor) to change the level of intracellular Ca^2+^, and examined the effect of exosomes from the treated MDA-MB-231 cells (Exo-A23187 or Exo-SKF) on angiogenesis of HUVECs. Furthermore, we investigated the effect of exosomes from STIM1-knockout MDA-MB-231 cells (Exo-STIM1-KO) on angiogenesis of HUVECs in vitro and Matrigel plug assay in vivo.

## Results

### Exosomes from MDA-MB-231 breast cancer cells promote angiogenesis

**Fig. 1:**
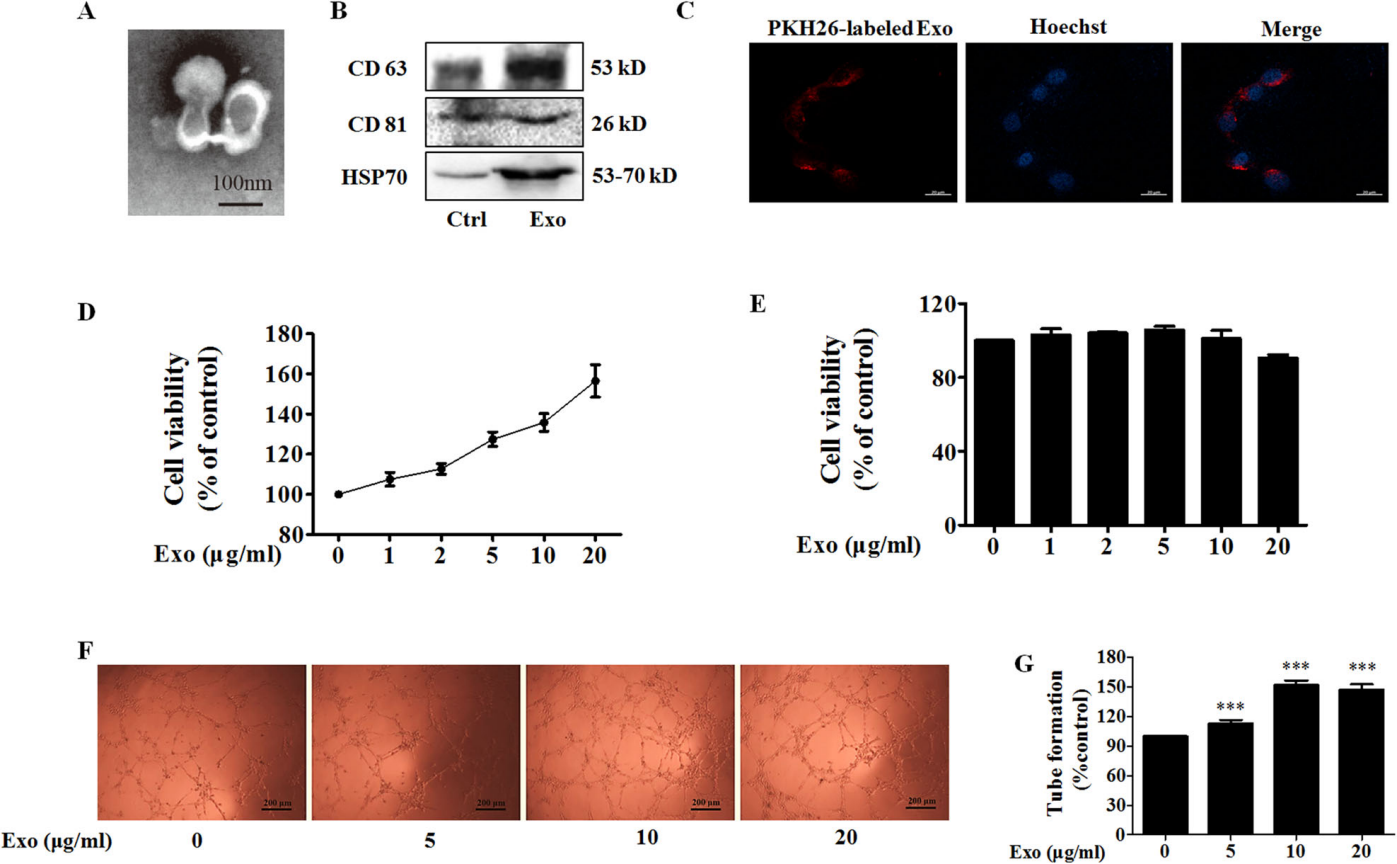
Exo promotes human umbilical vein endothelial cells (HUVEC) tube formation. **A** A representative transmission electron microscopic image of exosomes derived from MDA-MB-231 cells. **B** The protein markers of exosomes in Exo as determined by western blot. **C** The uptake of Exo labeled with PKH26 in HUVECs. **D** Effect of Exo on HUVEC viability. **E** Effect of Exo on MDA-MB-231 cell viability. **F** Exo promotes HUVEC tube formation. **G** Quantification of the results in (**F**), Data represent mean ± SEM from three independent experiments (*n* = 3). ***P < 0.001, compared with control (0 μg/ml).

The exosome is a major player in cell–cell communication, in which many materials such as miRNA, RNA, and proteins can be effectively transferred from the donor cells to the recipient cells^11^. We selected the triple-negative breast cancer MDA-MB-231 cells to study the effect of exosomes from cancer cells on angiogenesis in HUVECs. The typical cup-shaped morphology with a size range of 30–100 nm was observed under a transmission electron microscope (Fig. 1A). CD63, CD81, and HSP70, all of which are known as exosome markers^12^, were observed in MDA-MB-231 cells, and these MDA-MB-231 cells-derived exosomes (Exo) (Fig. 1B).

To study the biological function of exosomes from breast cancer on endothelial cells, we treated HUVECs with Exo. When Exo labeled with PKH26 (red) was incubated with HUVECs, the effective uptake of Exo by HUVECs was observed by electron microscopy (Fig. 1C). Next, we determined the effect of Exo on cell proliferation of MDA-MB-231 and HUVECs. Figure 1D, E showed that Exo increased proliferation of HUVECs in a concentration-dependent manner, but without effect on that of MDA-MB-231, after co-incubation for 24 h. These results revealed that MDA-MB-231-derived self exosomes (Exo) did not affect the proliferation of MDA-MB-231 cells, but increased the proliferation of endothelial cells. It is known that tumor growth and metastasis need glorious angiogenesis for nutrition provision. Therefore, we investigated the effects of Exo on HUVECs angiogenesis by measuring tube formation on Matrigel, after treatment for 24 h. Compared to the control group, Exo obviously increased HUVECs tube formation (Fig. 1F, G). The optimal Exo concentration of 10 μg/ml for angiogenesis was then used in subsequent experiments.

### Exo-A23187 promotes HUVEC proliferation, tube formation, and migration

**Fig. 2:**
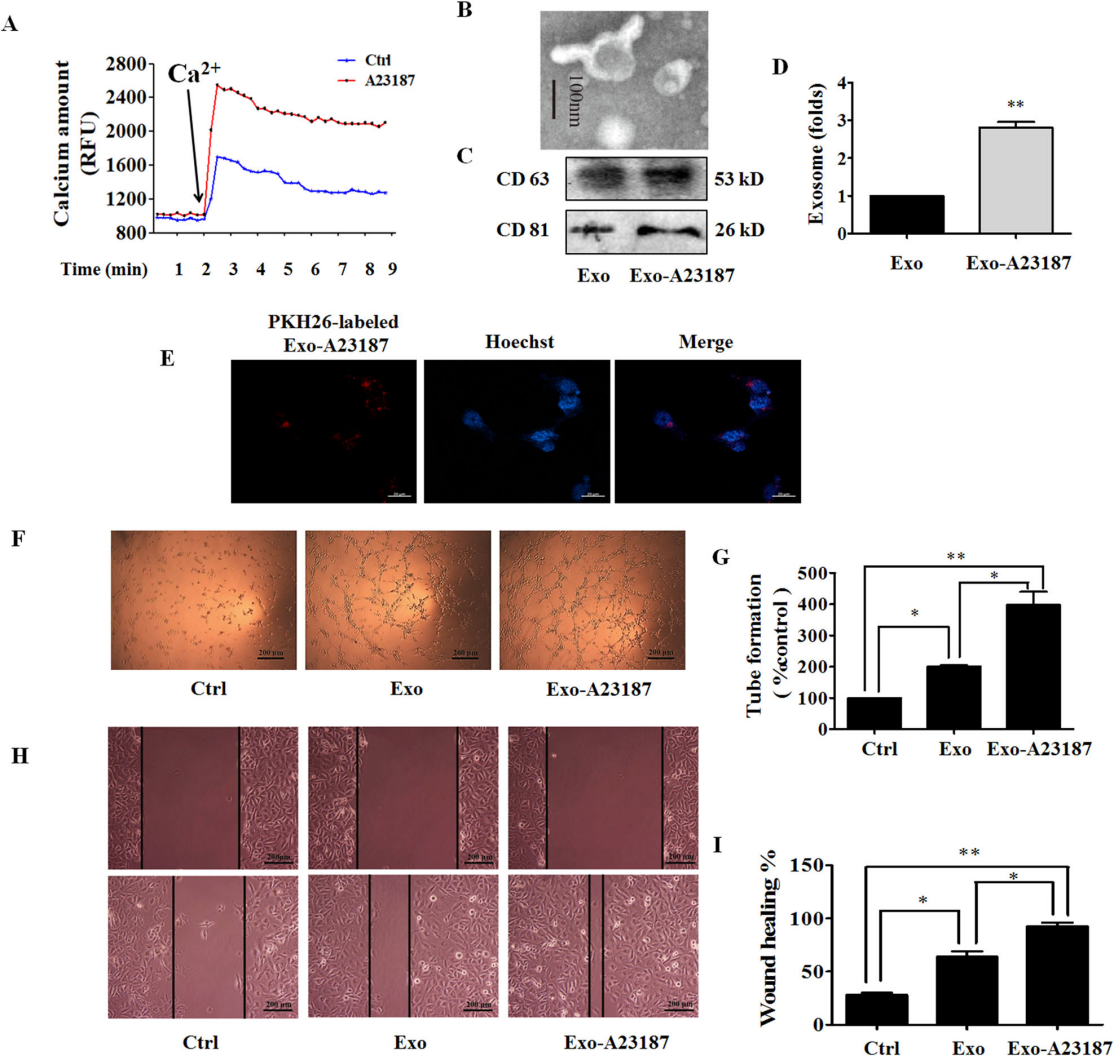
Exo-A23187 promotes human umbilical vein endothelial cells (HUVEC) proliferation, tube formation, and migration. **A** Effect of A23187 on intracellular Ca^2+^ level in MDA-MB-231 cells was determined with FluoForte Calcium Assay Kit. **B** Representative electron microscopic image of Exo-A23187. **C** The markers of exosomes in Exo or Exo-A23187 as determined by western blot. **D** A23187 increased the production of exosomes in MDA-MB-231 cells. **E** Images of intercellular trafficking of exosomes by isolated Exo-A23187 labeled with PKH26 in HUVECs. **F** Tube formation was increased in the Exo-A23187 group compared with the control and Exo group. **G** Quantification of the tube formation results in (**F**). **H** The migration of HUVECs was increased in the Exo-A23187 group compared with the control and Exo group. **I** Quantification of the HUVEC migration results in (**h**). Data represent mean ± SEM from three independent experiments (*n* = 3). *P < 0.05, **P < 0.01.

Tumorigenic pathways are associated with abnormal activation of Ca^2+^ channels^13^, and the increase of intracellular Ca^2+^ stimulates exosome secretion^14^. A23187 is a calcium ionophore which can be used to increase intracellular Ca^2+^ level in tumor cells. Because prolonged intracellular elevation of Ca^2+^ might result in cell death, we first examined the effect of A23187 on cell viability, apoptosis, and cell cycle. As a result, A23187 reduced cell viability, induced apoptosis, and G0/G1 cell cycle arrest at high concentrations, and elevated intracellular Ca^2+^ level in a concentration-dependent manner (Supplementary Fig. S1a–f). We also found that A23187 promoted the migration of MDA-MB-231 cells at non-cytotoxic concentrations of <200 nM (Supplementary Fig. S1g, h). These results are consistent with that prolonged intracellular elevation of Ca^2+^ can be cytotoxic, and intracellular Ca^2+^ alteration is involved in tumor progression and metastasis^15^. We chose the concentration of A23187 as 500 nM, and treating time as 6 h for the following experiments. The effect of A23187 on intracellular Ca^2+^ level in MDA-MB-231 cells was determined with FluoForte Calcium Assay Kit. Figure 2A showed that treatment of MDA-MB-231 with A23187 in the presence of extracellular Ca^2+^ led to an increase of intracellular Ca^2+^. To determine the effect of intracellular calcium increase on the release of exosomes, MDA-MB-231 cells were treated with A23187. The characterized exosomes were found by using transmission electron microscopy in Exo-A23187 with a uniformly cup-shaped morphology within 30–100 nm as diameter (Fig. 2B). A23187-treated MDA-MB-231 cells showed a significant increase in the number of exosomes compared to normal MDA-MB-231 cells (Fig. 2C, D), and HUVECs also exhibited an efficient uptake of Exo-A23187 (Fig. 2E). Next, we determined the effects of Exo and Exo-A23187 on in vitro angiogenesis of HUVECs. In the tube-formation assay, HUVECs were incubated with equivalent concentration (10 μg/ml) of Exo and Exo-A23187 for 24 h and seeded onto matrigel. The results showed that the Exo-A23187 significantly increased tube formation by HUVECs compared to the Exo-treated group, with the tube length elevated by 197 ± 8% (Fig. 2F, G). Cell migration was assessed by the scratch wound-healing assay. The migration ability of HUVECs was enhanced in the Exo group compared with the control, and the Exo-A23187 group was further enhanced compared with Exo by 28 ± 4% (Fig. 2H, I). These results demonstrated that A23187 elevated intracellular calcium and therefore increased the release of exosomes from MDA-MB-231 cells, resulting in an increase of HUVEC tube formation and migration.

### Exo-SKF suppresses HUVEC tube formation and migration

**Fig. 3:**
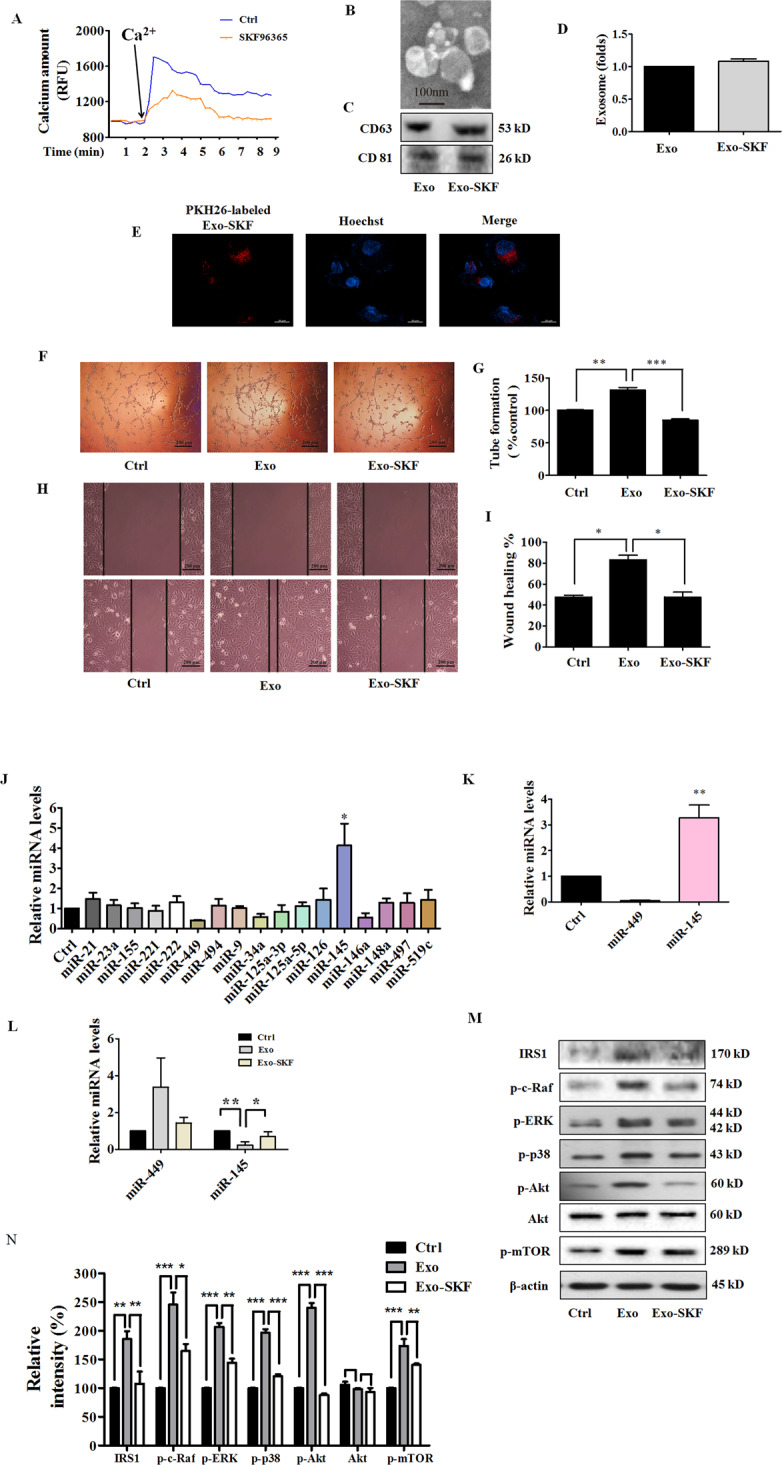
Exo-SKF inhibits human umbilical vein endothelial cells (HUVEC) tube formation and migration through the miR-145 and IRS1 pathways. **A** Representative time-course recording of intracellular Ca^2+^ fluorescence showing the inhibitory effect of SKF96365 on Ca^2+^ influx in MDA-MB-231 cells, which was determined with FluoForte Calcium Assay Kit. **B** Representative electron microscopic image of Exo-SKF. MDA-MB-231 cells were treated with 10 μM of SKF96365. **C** The markers of exosomes in Exo and Exo-SKF, as determined by western blot. **D** SKF96365 did not affect the production of exosomes in MDA-MB-231 cells. **E** The uptake of Exo-SKF in HUVECs. **F** The Exo-SKF96365 inhibits tube formation in HUVECs. **G** Quantification of the results in (**F**). **H** Exo-SKF96365 reduces HUVEC migration. **I** Quantification of the results in (**H**). **J** Changes of miRNA level in MDA-MB-231 cells were detected by RT-qPCR after treatment with SKF96365. **K** The relative levels of miR-145 and miR-449 in Exo-SKF. **L** The relative levels of miR-145 and miR-449 in HUVECs after Exo or Exo-SKF treatment. **M** The expression of IRS1 and phosphorylation of c-Raf, ERK, p38, Akt, and mTOR in HUVECs after Exo or Exo-SKF treatment. **N** Quantification of the results in (**M**). Data represent mean ± SEM from three independent experiments (*n* = 3). *P < 0.05, **P < 0.01, and ***P < 0.001.

Inhibition of tumor angiogenesis might halt tumor progression. The above results showed that exosomes from breast cancer cells with higher intracellular Ca^2+^ promoted HUVEC angiogenesis. We hypothesized that exosomes from cancer cells with lower intracellular Ca^2+^ might exhibit an anti-angiogenic effect. Then we used SKF96365, a pharmacological store-operated Ca^2+^ influx inhibitor, to reduce intracellular Ca^2+^ level. We found that SKF96365 reduced the cell viability and augmented cell apoptosis at concentrations of 20 μM and 50 μM (Supplementary Fig. S2a–c). Therefore, we chose the non-cytotoxic but effective concentration of 10 μM for the following experiments. We first examined the inhibitory effect of SKF96365 on Ca^2+^ entry in MDA-MB-231 cells. MDA-MB-231 were stained with FluoForte^TM^ dye-loading solution. The subsequent introduction of CaCl_2_ to the extracellular solution resulted in the elevation of intracellular Ca^2+^ from baseline, and pretreatment of SKF96365 reduced the intracellular level of Ca^2+^ (Fig. 3A). Afterward, we isolated Exo-SKF, and examined the Exo-SKF morphology and the exosome-specific marker proteins by using electron microscopy and western blot (Fig. 3B, C). Treatment of SKF96365 did not significantly change the release of exosomes from MDA-MB-231 cells (Fig. 3D). We also visualized the transport of Exo-SKF into HUVECs. PKH26 (red)-labeled Exo-SKF was incubated with HUVECs after staining the cells with Hoechst, and the PKH26 signals and Hoechst signals were detected under a fluorescent microscope. We found that almost all the recipient HUVECs revealed red signal, suggesting that the uptake of Exo-SKF by HUVECs was efficient (Fig. 3E). And the treatment of Exo-SKF significantly suppressed the tube-formation ability of HUVECs compared with Exo (Fig. 3F, G). In addition, the migration ability of HUVECs was suppressed in the Exo-SKF group compared with Exo treatment only (Fig. 3H, I). These results demonstrated that intracellular Ca^2+^ regulated the release of exosomes and affected tumor angiogenesis.

### Exo-SKF affects the levels of miR-145 and miR-449

Exosomes released from tumor cells contain miRNAs which could even affect the environment surrounding the tumor. miRNAs work via translational inhibition or degradation of their target mRNAs to downregulate gene expression. Thus, we determined the levels of miRNAs relating to angiogenesis in SKF96365-treated MDA-MB-231 cells. We measured expression of 17 human miRNAs including miR-21, miR-23a, miR-155, miR-221, miR-222, miR-449, miR-494, miR-9, miR-34a, miR-125a-3p, miR-125a-5p, miR-126, miR-145, miR-146a, miR-148a, miR-497, and miR-519c, which act as pro-angiogenic or anti-angiogenic factors^16–21^ or are dysregulated in breast cancer^22–25^. MDA-MB-231 cells were treated with SKF96365 for 24 h. We found a significantly higher expression of miR-145 and lower expression of miR-449 in SKF96365-treated MDA-MB-231 cells (Fig. 3J). Furthermore, we isolated Exo from the SKF96365-treated MDA-MB-231 cells and found that miR-145 was highly expressed and miR-449 barely detected (Fig. 3K). Similar results were also found in Exo-SKF-treated HUVECs. The miR-449 level was elevated in Exo-treated group compared to the control, and Exo-SKF alleviated such effect. In contrast, the miR-145 level was reduced in Exo-treated group, and Exo-SKF enhanced such effect (Fig. 3L). Since miR-145 affected more obviously than miR-449, we focused on miR-145 in the subsequent experiments.

### Exo-SKF suppresses insulin receptor substrate 1 (IRS1) signaling pathway

miRNAs regulate post-transcriptional expression by gene silencing. As the predicted target of miR-145^26^, IRS1 activates phosphatidylinositol 3-kinase (PI3K)/Akt pathway, and mitogen-activated protein kinase (MAPK) pathway to promote endothelial cell proliferation, migration, as well as vascular permeability^27^. Thus, we investigated the effect on IRS1 signaling pathways by evaluating the changes of c-Raf, extracellular signal regulated-protein kinase (ERK), p38, Akt, and mammalian target of rapamycin (mTOR). HUVECs were treated with Exo or Exo-SKF for 24 h, and cells were lysed for western blot. As shown in Fig. 3M, N, we observed upregulation of IRS1 and its downstream signal proteins such as c-Raf, ERK, p38, Akt, and mTOR phosphorylation in HUVECs treated with Exo, but downregulation in those treated with Exo-SKF.

### Exo-STIM1-KO suppresses HUVEC tube formation

**Fig. 4:**
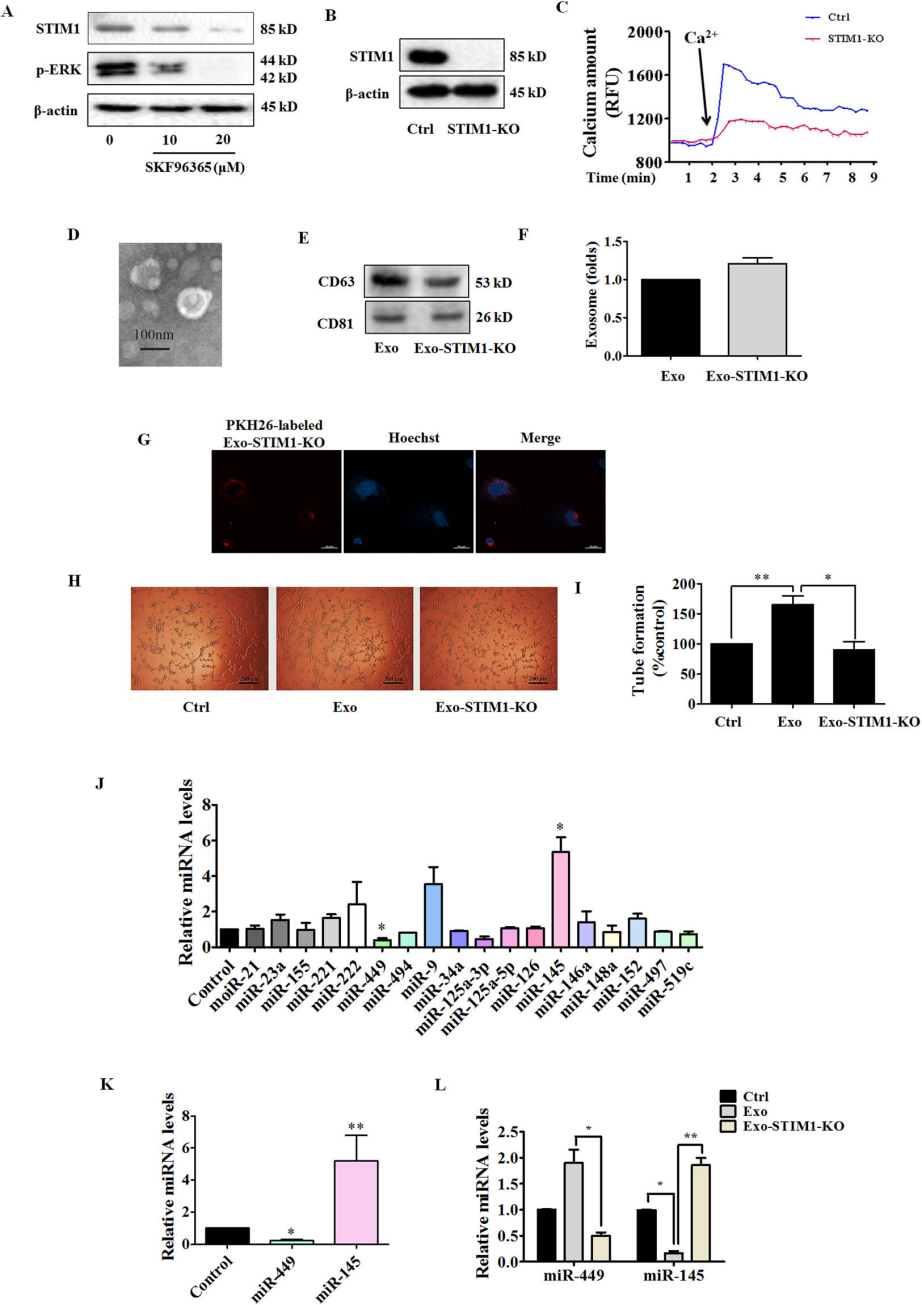
Exo-STIM1-KO suppresses human umbilical vein endothelial cells (HUVEC) tube formation and increases miR-145 level. **A** Expression of STIM1 and phosphorylation of ERK in SKF96365-treated MDA-MB-231 cells. **B** Expression of STIM1 in STIM1-KO MDA-MB-231 cells. **C** Representative time-course recording of intracellular Ca^2+^ fluorescence in STIM1-KO-MDA-MB-231 cells. **D** Representative electron microscopic image of Exo-STIM1-KO. **E** The markers of exosomes in Exo-STIM1-KO, as determined by western blot. **F** The amount of Exo-STIM1-KO showed no significant change compared with Exo. **G** The uptake of Exo-STIM1-KO in HUVECs. HUVECs were cultured with PKH26-labeled Exo-STIM1-KO. **H** Tube formation was inhibited by Exo- STIM1-KO. **I** Quantification of the results in (**H**). **J** Relative levels of miRNAs in STIM1-silencing MDA-MB-231 cells. **K** The levels of miR-145 and miR-449 in Exo-STIM1-KO. **L** The levels of miR-145 and miR-449 in HUVECs after treatment with Exo and Exo-STIM1-KO. Data represent mean ± SEM from three independent experiments (*n* = 3). *P < 0.05, **P < 0.01.

STIM1 plays an essential role in Ca^2+^ mobilization and signaling^28^. As the ER Ca^2+^ sensor, STIM1 controls plasma membrane Ca^2+^ channels to regulate Ca^2+^ entry. After MDA-MB-231 cells were treated with SKF96365 for 24 h, the expression of STIM1 and phosphorylation of ERK were downregulated (Fig. 4A). To investigate the role of STIM1 in the reduction of angiogenesis by Exo-SKF, we edited exon 4 of STIM1 locus (ENSG00000167323) in MDA-MB-231 cells by CRISPR/Cas9 gene editing to generate the Exo-STIM-KO, and examined the anti-angiogenic effects on HUVECs. A schematic diagram of gRNA-targeting exon 4 of the STIM1 gene is shown in Supplementary Fig. S3a. We first confirmed the sequence of exon 4 in STIM1 in MDA-MB-231 cells with Sanger sequencing, which revealed that MDA-MB-231 cells carried 1-bp deletion at the gRNA-targeting region (Supplementary Fig. S3b). After Sanger sequencing validation, we obtained STIM1-knockout MDA-MB-231 cells (STIM1-KO). To further determine the effectiveness of CRISPR/Cas9, a western blot was carried out to detect STIM1 expression. STIM1-CRISPR/Cas9 gene-edited MDA-MB-231 cells showed no expression of STIM1 (Fig. 4B). The lack of STIM1 led to a significant drop in calcium amount, which was assessed in the presence of additional Ca^2+^ (Fig. 4C). The characteristic morphology of Exo-STIM1-KO was observed under an electron microscope (Fig. 4D), and both CD63 and CD81 were found in Exo-STIM1-KO (Fig. 4E). An equal number of MDA-MB-231 and STIM1-deficient MDA-MB-231 cells were incubated in culture dishes, and then the exosomes were isolated respectively. The amount of Exo-STIM1-KO did not significantly change compared with Exo (Fig. 4F). Exo-STIM1-KO was also labeled with PKH26 red dye, and the uptake by HUVECs was observed (Fig. 4G). We then examined whether Exo-STIM1-KO could affect angiogenesis. HUVECs were incubated with Exo or Exo-STIM1-KO for 24 h. As expected, Exo-STIM1-KO repressed the tube formation of HUVECs compared to Exo treatment only (Fig. 4H, I). Therefore, our results suggest that STIM1-deficient MDA-MB-231 cells inhibit HUVEC angiogenesis through the exosomes.

### Knockout of STIM1 upregulates miR-145

To further elucidate the molecular mechanism of the anti-angiogenic effect induced by Exo-STIM1-KO, we also analyzed the levels of 17 human miRNAs in STIM1-deficient MDA-MB-231 cells, Exo-STIM1-KO, and HUVECs treated with Exo-STIM1-KO. Among 17 miRNAs, miR-145 was upregulated and miR-449 was downregulated in STIM1-deficient MDA-MB-231 cells, Exo-STIM1-KO, and Exo-STIM1-KO-treated HUVECs (Fig. 4J–L). Moreover, miR-145 changed more obviously than miR-449. These results are consistent with those in Exo-SKF-treated HUVECs.

### Exosomal miR-145 from STIM-KO-MDA-MB-231 cells targets IRS1

**Fig. 5:**
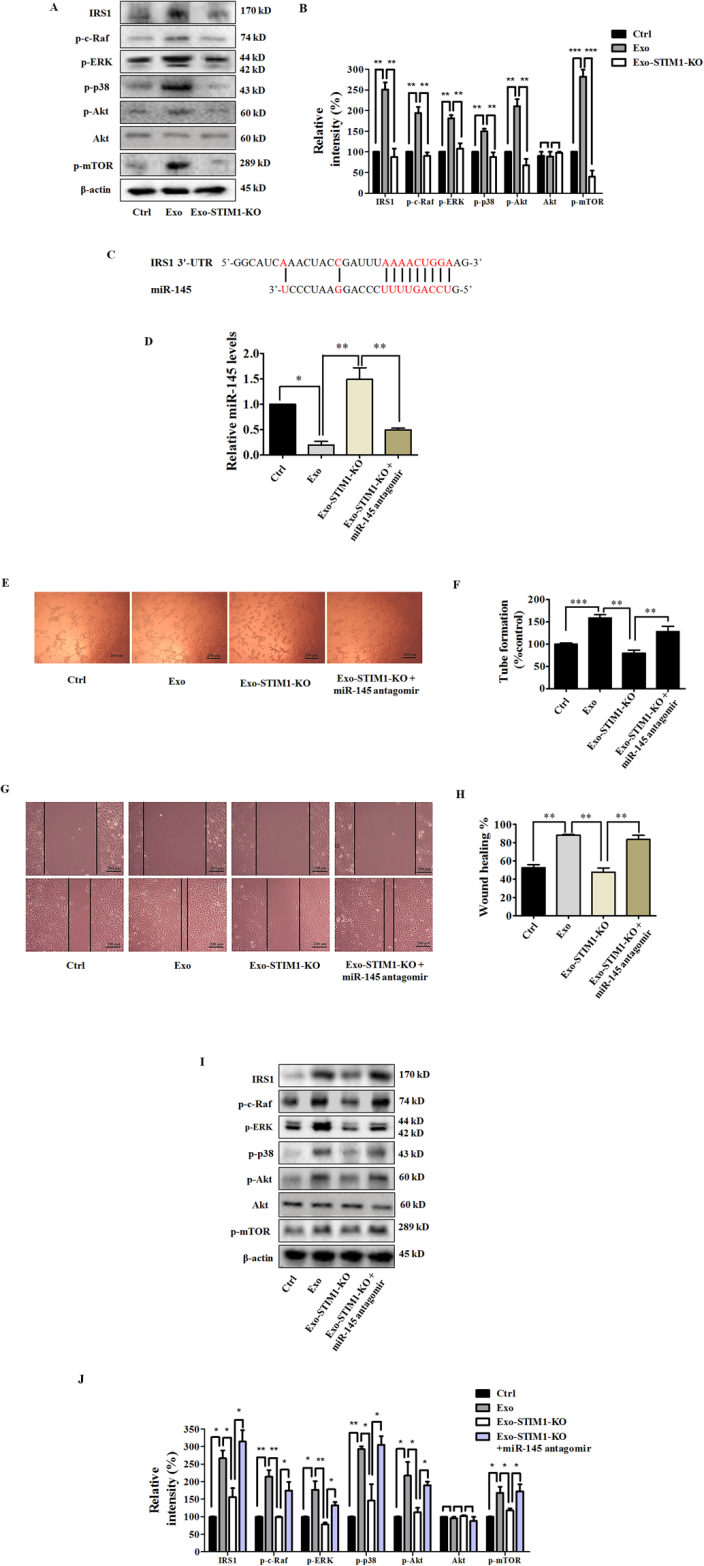
Exo-STIM1-KO miR-145 inhibits angiogenesis through targeting IRS1 signal pathway in human umbilical vein endothelial cells (HUVECs). **A** Western blot analysis of IRS1 and its downstream signal proteins in HUVECs. **B** Quantification of the results in (**A**). **C** Schematic diagrams of miR-145 binding sites in the 3’-UTR of IRS1. **D** The levels of miR-145 in HUVECs after various treatments. **E** Images of tube formation of HUVECs after various treatments. **F** Quantification of the results in (**E**). **G** Representative migration of HUVECs after various treatments. **H** Quantification of the results in (**G**). **I** Western blot analysis of IRS1 and the downstream signal proteins in HUVECs treated with Exo-STIM1-KO plus/or miR-145 antagomir. **J** Quantification of the results in (**I**). Data represent mean ± SEM from three independent experiments (*n* = 3). *P < 0.05, **P < 0.01, and ***P < 0.001.

We subsequently examined the effect of exosomal miR-145 from STIM1-KO-MDA-MB-231 cells on the IRS1 pathway, which is the key pathway regulating tumor angiogenesis. HUVECs were incubated with Exo or Exo-STIM1-KO for 24 h and collected for western blot. As shown in Fig. 5A, B, IRS1 was increased by Exo, but reduced by Exo-STIM1-KO. Furthermore, phosphorylation of IRS1 pathway proteins such as Raf, ERK, p38, Akt, and mTOR was also elevated by Exo, but repressed by Exo-STIM1-KO. These results are consistent with Exo-SKF-treated HUVECs. Since several reports have shown that miR-145 directly targets IRS1^29–32^, and base-pairing complement suggests that miR-145 binds on IRS1 3’-untranslated regions (3’-UTR) (Fig. 5C), we hypothesized that miR-145 might target IRS1 to play a pathological role in the angiogenesis process. To confirm this hypothesis, we used miR-145 antagomir to examine the angiogenic effect of Exo-STIM1-KO in HUVECs. HUVECs were treated with Exo, Exo-STIM1-KO, Exo-STIM1-KO plus miR-145 antagomir, or miR-145 antagomir for 24 h. The results showed that the Exo-STIM1-KO elevated level of miR-145 compared to the Exo group, while Exo-STIM1-KO plus miR-145 antagomir decreased the level of miR-145 compared to the Exo-STIM1-KO group (Fig. 5D).

Then HUVECs were treated with different exosomes for 24 h, and the tube formation and cell migration abilities were investigated. Upregulation of tube formation by Exo was suppressed by Exo-STIM1-KO, and Exo-STIM1-KO plus miR-145 antagomir reversed this inhibition (Fig. 5E, F). As shown in Fig. 5G, H, cell migration was significantly suppressed in the Exo-STIM1-KO group in comparison with the Exo group. Conversely, Exo-STIM1-KO plus miR-145 antagomir group enhanced the migration ability of HUVECs. These results suggested that miR-145 plays an important role in endothelial cell migration mediated by Exo-STIM1-KO.

Next, we examined the expression of IRS1 pathway proteins in HUVECs after incubation with different exosomes for 24 h. miR-145 antagomir upregulated the expression of IRS1, and activated the IRS1-dependent Akt/mTOR, Raf/ERK, and p38 MAPK. In addition, Exo-STIM1-KO plus miR-145 antagomir obviously elevated the expression of IRS1 and activated its downstream molecules which had been suppressed by Exo-STIM1-KO (Fig. 5I, J).

### Exo-STIM1-KO prevents breast cancer angiogenesis in vivo

**Fig. 6:**
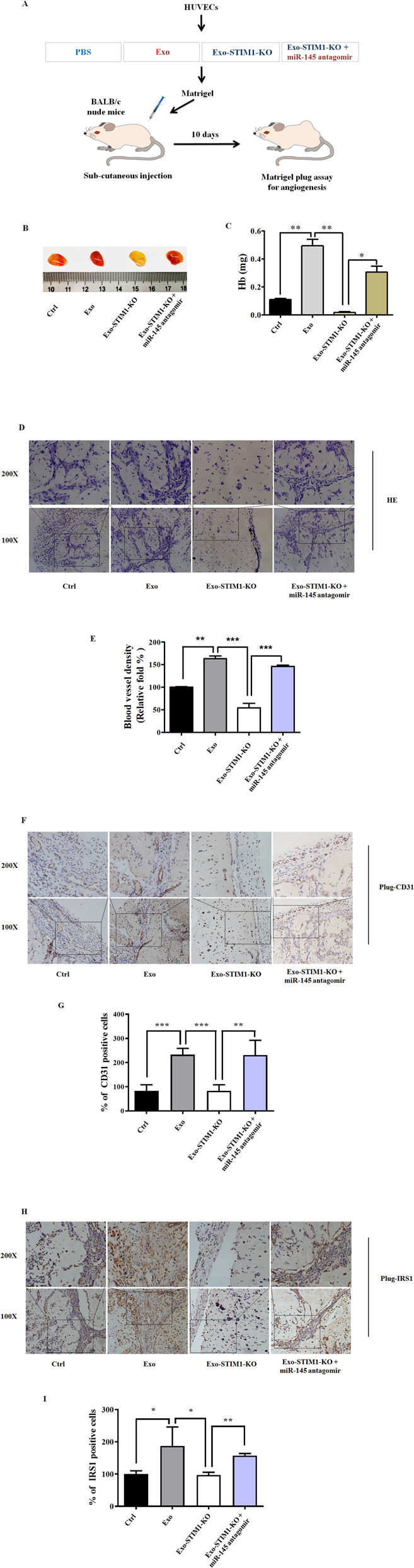
Exo-STIM1-KO inhibits angiogenesis in nude mice Matrigel plug angiogenesis models. **A** Schematic description of the in vivo angiogenesis experiment. **B** Exo-STIM1-KO significantly reduced angiogenesis. Representative photographs of angiogenesis in the nude mice are shown. **C** Plug hemoglobin amount representing vascularity. **D** H&E staining of Matrigel plug sections. **E** Quantification of the results in (**D**). **F** Representative immunohistochemical staining of CD31. **G** Quantification of the results in (**F**). **H** Representative immunohistochemical staining of IRS1. **I** Quantification of the results in (**H**). Data represent mean ± SEM from three independent experiments (*n* = 3). *P < 0.05, **P < 0.01, and ***P < 0.001.

To further determine the therapeutic potential of Exo-STIM1-KO in vivo, we performed an in vivo Matrigel plug assay to detect the newly formed blood vessels in the transplanted gel plugs in BALB/c nude mice (Fig. 6A). The hemoglobin content, which represents new vessel formation, was significantly increased in Exo-treated mice compared with the control group but was reduced in the Exo-STIM1-KO group than the Exo-treated group. In contrast, the hemoglobin concentration in the plugs containing Exo-STIM1-KO plus miR-145 antagomir was higher than the Exo-STIM1-KO group (Fig. 6B, C). H&E staining assay revealed that Exo dramatically increased plug vascularization compared to control, and Exo-STIM-KO suppressed plug neovessel formation. However, Exo-STIM-KO plus miR-145 antagomir reversed the Exo-STIM-KO induced angiogenesis attenuation (Fig. 6D). The blood vessel density which represents the relative tube length of the neovessels in Matrigel plugs was quantified (Fig. 6E). Moreover, immunohistochemistry analysis confirmed that the CD31 (a marker of the formation of new vessels) positive cells in Exo-STIM1-KO-treated mice were significantly fewer than the Exo-treated mice, but increased when miR-145 antagomir was added (Fig. 6F, G). In addition, the expression of IRS1 was also lower in the Exo-STIM1-KO-treated mice compared with the Exo group but was upregulated in Exo-STIM1-KO plus miR-145 antagomir-treated mice (Fig. 6H, I).

## Discussion

Exosomes are known as potent cell–cell messenger. Various cell types have the ability to release exosomes, including immune cells, epithelial cells, and tumor cells. The mechanisms involved in the entry of exosomes in recipient cells include three main routes, such as receptor/ligand signaling, fusion, phagocytosis/endocytosis^33^. In this study, we found that PKH26-labeled Exo, Exo-A23187, Exo-SKF, and Exo-STIM1-KO from MDA-MB-231 cells (donor cells) transferred to the HUVECs (recipient cells). Exosomes contribute to carcinogenesis by the transport of oncogenic lipid, proteins, and nucleic acids, and the other bioactive molecules^34^. In this study, we found that MDA-MB-231-derived exosomes promoted tube formation in HUVECs.

Angiogenesis is a fundamental process involved in carcinogenesis. Steps toward angiogenesis include endothelial cell migration and proliferation, vascular tube formation, anastomosis of newly formed tubes^35^. An increase of intracellular Ca^2+^ stimulates exosome secretion^14^. However, the effects of exosomes from the breast cancer cells with elevated or decreased Ca^2+^ conditions on angiogenesis have not been reported yet. Therefore, we treated MDA-MB-231 cells with calcium ionophore or inhibitor of store-operated Ca^2+^ channel, and isolated exosomes to investigate their effects on angiogenesis in HUVECs. Our results showed that treatment of MDA-MB-231 cells with A23187 or SKF96365 in the presence of extracellular Ca^2+^ led to an increase or decrease of intracellular Ca^2+^, respectively. We also found that Exo-A23187 promoted HUVECs migration and tube formation, and Exo-SKF inhibited them. These results suggested that change of Ca^2+^ level influenced exosome release from triple-negative breast cancer MDA-MB-231 cells, and therefore affected angiogenesis of HUVECs.

STIM1 is a critical regulator of Ca^2+^ mobilization. Once ER Ca^2+^ is depleted, the ER Ca^2+^ sensor STIM1 accumulates in a junctional ER in close apposition to the plasma membrane to activate the plasma membrane pore-forming unit Orai1, which induces SOCE^36^. Therefore, we deleted the STIM1 gene by CSISPR/Cas9, and found the intracellular Ca^2+^ was decreased in the STIM1-silencing MDA-MB-231 cells, the characteristic morphology and markers of exosomes were also observed. The exosome amounts did not change obviously.

miRNAs are highly conserved small noncoding RNAs that induce post-transcriptional gene silencing canonically by binding mRNA. miRNA/mRNA interaction hinders protein synthesis and initiates mRNA degradation^37^. Some miRNAs are known to be involved in tumorigenesis and tumor angiogenesis. For example, miR-34a is a tumor suppressor that is frequently downregulated in a number of tumor types, and ectopic expression of miR-34a in head and neck squamous cell carcinoma (HNSCC) cell lines inhibited tumor growth and tumor angiogenesis in vivo when introduced into SCID mouse xenograft models^38^; miR-519c is a pivotal regulator of tumor angiogenesis^39^; modulation of miR-126 expression can disrupt angiogenesis and vascular integrity^40^. Some other miRNAs such as miR-21, miR-221, and miR-222 are highly expressed in the endothelial cells^41^. In the present study, we demonstrated that level of miR-145 increased in MDA-MB-231 cells treated with SKF, STIM1-KO, Exo-SKF, and Exo-STIM1-KO, as well as in HUVECs treated with Exo-SKF and Exo-STIM1-KO. It was reported that miR-145 is downregulated in breast cancer cancer^42^. These results support that Ca^2+^ sensor STIM1 might influence tumor angiogenesis through regulating exosomal miR-145.

The pathway of insulin-like growth factor I (IGF-I)/IRS1/Ras mediates vascular endothelial growth factor (VEGF) production, which is crucial in angiogenesis. IR is expressed in endothelial cells as well as in cancer cells^43^. Insulin stimulation increases the tyrosine phosphorylation of IRS1 and IRS2^44^. miR-145 has been repeatedly reported to be a tumor suppressor, and to target gene IRS1 and therefore inhibit its protein expression^29^. Through base-pairing complement, we also confirmed that IRS1 is the potential target of miR-145. As expected, Exo promoted the IRS1 expression in HUVECs, which was attenuated by treatment with Exo-SKF or Exo-STIM1-KO. Tyrosin-phosphorylated IRS1 activates PI3K/Akt and Ras/Raf/MAPK pathways^45,46^, which mediate endothelial cell proliferation, migration, survival, and vascular permeability^47,48^. In this study, we found that the phosphorylation of Raf, ERK, p38, Akt, and mTOR in HUVECs was increased by treatment with Exo, but was attenuated by concomitant treatment with SKF (Exo-SKF) and STIM1-KO (Exo-STIM1-KO). These results suggest that miR-145 might target IRS1 and therefore inhibit the angiogenesis of HUVECs via regulating IRS1/PI3K/Akt/mTOR and IRS1/Raf/ERK pathways.

We next used miR-145 antagomir to demonstrate whether Exo-STIM1-KO miR-145 could reduce angiogenesis through directly repressing IRS1. The level of miR-145 in Exo-STIM1-KO plus miR-145 antagomir group is lower than that in the Exo-STIM1-KO group. Suppression of miR-145 in recipient HUVECs by miR-145 antagomir transfection abolished the anti-angiogenic effect of Exo-STIM1-KO in HUVECs, suggesting that miR-145 might play pivotal roles in the Exo-STIM1-KO attenuated tumor angiogenesis effect.

The in vivo Matrigel plug assay is widely used to evaluate the in vivo angiogenic potential^49^. To date, there has been no report on the anti-angiogenic effects of exosomes derived from STIM1-KO-tumor cells. By using in vivo Matrigel plug assay, we found that Exo-STIM1-KO significantly inhibited Exo-induced angiogenesis. Consistently, immunohistochemical analysis showed a reduction of expression of IRS1 and angiogenesis biomarker CD31, which was reversed by the addition of miR-145 antagomir. These results confirmed that the exosomal miR-145 from STIM-KO-MDA-MB-231 cells attenuated angiogenesis by targeting IRS1.

**Fig. 7:**
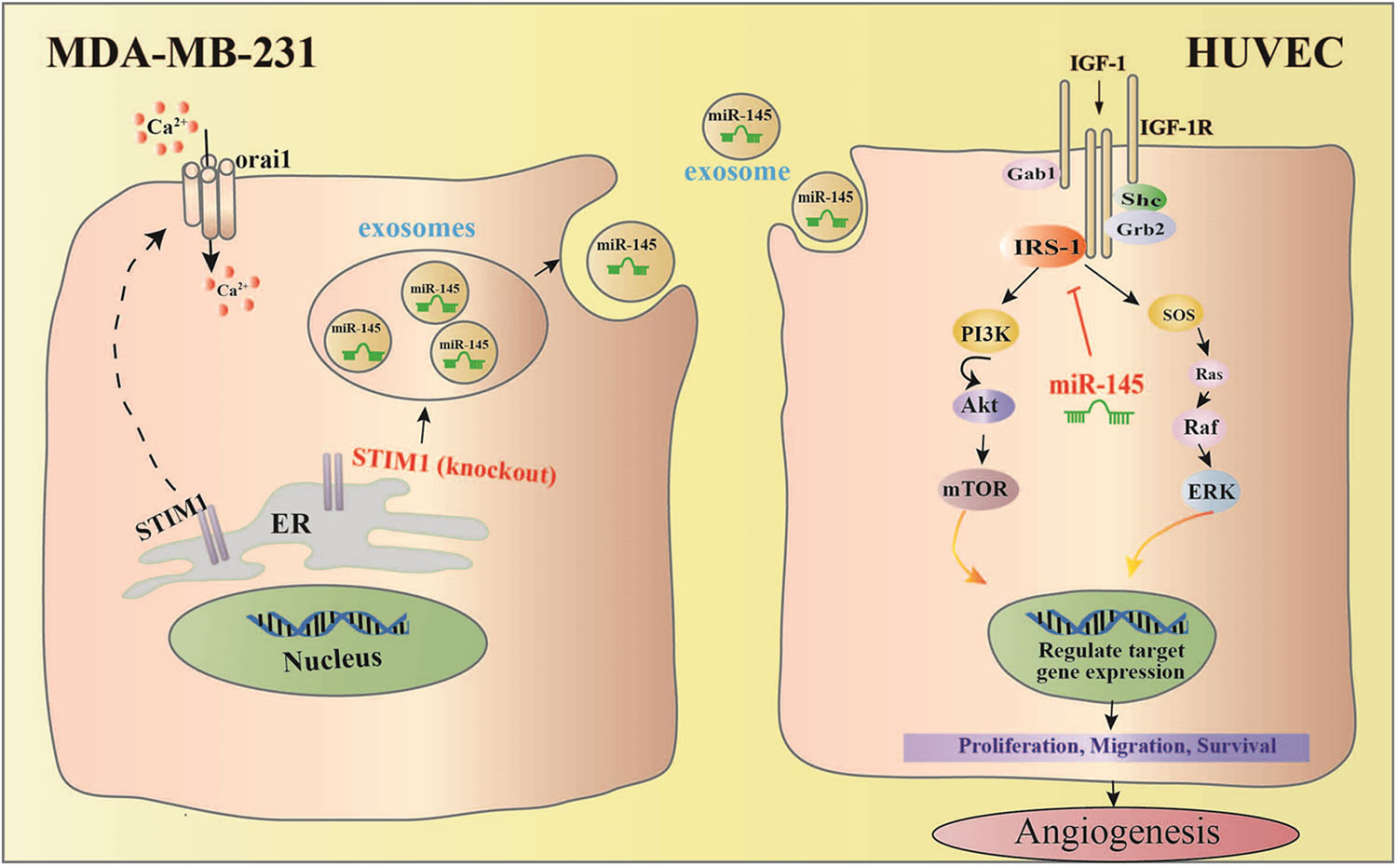
Exosomes from STIM1-KO-MDA-MB-231 breast cancer cells contain higher levels of miR-145, which targets IRS1 and its downstream signal to suppress tube formation of vascular endothelial cells, resulting in anti-angiogenesis effect. Predicted anti-angiogenic mechanism of exosomal miR-145 from STIM1-KO-MDA-MB-231 cells via targeting IRS1.

Overall, our studies have demonstrated that exosomes from breast cancer cells with a lower level of Ca^2+^ contain more miR-145, which targets IRS1 to exhibit an anti-angiogenic effect (Fig. 7). Our results suggest that the reduction of Ca^2+^ level in cancer cells might contribute to anti-angiogenic tumor therapy.

## Materials and methods

### Cell culture and reagents

MDA-MB-231 cells were purchased from the cell bank of the Chinese academy of sciences (Shanghai, China). No contamination of mycoplasma was confirmed by PCR test. Cells were cultured in DMEM supplemented with 10% fetal bovine serum (FBS), penicillin and streptomycin at 37 °C in a humidified atmosphere containing 5% CO_2_.

DMEM, FBS, the enhanced chemiluminescence (ECL) reagent, and Total exosome isolation reagents were purchased from Thermo Fisher Scientific (4478359; Waltham, MA, USA). Matrigel was purchased from BD Biosciences (San Josè, CA, USA). PKH26 Red fluorescent cell linker kit was purchased from Sigma Chemicals (St. Louis, MO, USA). TRIzol reagent was obtained from Invitrogen (Carlsbad, CA). The antibodies specific for phospho-c-Raf (#9427), phospho-ERK (#4377), phospho-p38 (#9211), phospho-Akt (#9271), Akt (#9272), phospho-mTOR (#2971), STIM1 (#A5668) β-actin (#4967), and the horseradish peroxidase-conjugated goat anti-rabbit secondary antibody were purchased from Cell Signaling Technology, Inc. (Danvers, MA, USA). The anti-IRS1 (ab40777) and anti-CD31 (ab28364) antibodies were obtained from Abcam (Cambridge, MA, USA). The exosome-specific primary antibodies including CD9, CD63, CD81, and HSP70 were obtained from System Biosciences (EXOAB-KIT-1, Palo Alto, CA, USA).

### Cell proliferation assay

Cell proliferation was assessed using an MTT assay as we previously reported^50^ with a small modification. Briefly, cells were seeded onto 96-well plates and cultured with exosomes from MDA-MB-231 cells for 24 h, and then MTT was added to each well. After 4 h of incubation, the produced formazan was dissolved in DMSO, and OD at 490 nm was monitored using microplate reader iMark (BIO-RAD, Hercules, CA, USA).

### Isolation of exosomes

MDA-MB-231 cells were seeded at a density of 2 × 10^6^ cells/100-mm dish and cultured for 6 h or 24 h without or with the treatment of A23187 (500 nM) or SKF96365 (10 μM) in serum-free medium. Exo, Exo-A23187, Exo-SKF96365, and Exo-STIM1-KO in cell culture supernatants were isolated by use of total exosome isolation reagents as described by the manufacturer. Exosome protein amount was quantified using the Pierce^TM^ BCA protein assay kit (23225; Thermo Fisher Scientific, Waltham, MA, USA).

### Wound-healing assay

HUVECs were cultured on 24-well plates (2 × 10^5^ cells/well) in DMEM medium. Wounds were made using a sterile 10-µl pipette tip. After the cellular debris was removed by gently washing with PBS, the cells were cultured in high glucose serum-free DMEM with various exosomes at 37 °C with 5% CO_2_. At least four images of the scraped area were captured using phase-contrast microscopy after 24 h treatment, and cell migration distances were determined using ImageJ software.

### Intracellular Ca^2+^ measurement

Intracellular Ca^2+^ level was determined with the FluoForte Calcium Assay Kit (Enzo Life Sciences, Ann Arbor, MI, United States). Briefly, MDA-MB-231 or STIM1-knockout MDA-MB-231 cells were cultured on 96 poly-D-lysin-coated glass bottom plate, stained with FluoForte^TM^ dye-loading solution for 1 h, and then treated with A23187 or SKF96365 for 1 h. After the addition of CaCl_2_ (8 mM), the fluorescence was determined with a multilabel plate reader VICTOR (Perkin Elmer, Waltham, MA, USA) at Ex = 485 nm/Em 535 nm at 20-s interval.

### Tube-formation assay

HUVECs (1 × 10^5^ cells/well) were treated with exosomes derived from MDA-MB-231 cells after various treatments for 24 h and then plated on Matrigel^TM^ Matrix (50 μl/well) (BD Biosciences, San Josè, CA, USA) in 96-well plates for 6 h at 37 °C in a 5% CO_2_ humidified incubator. Tube formation was observed and imaged with phase-contrast microscopy. For quantification, total tubular length and branch points per well were determined using ImageJ software.

### Protein extraction and western blot

Western blot analysis was carried out as we previously reported^51^ with a small modification. Cells were collected, and the protein concentration of each sample was determined by the BCA protein assay kit. Equal amount of protein was separated by sodium dodecyl sulfate-polyacrylamide gel electrophoresis (SDS-PAGE) and transferred to the PVDF membrane. After being blocked with 5% skim milk, the membranes were incubated with each primary antibody, and then the horseradish peroxidase-conjugated secondary antibody. The signals were detected with ChemiDoc^TM^ XRS + System (BIO-RAD, Hercules, CA, USA) after exposure to ECL reagent.

### Fluorescent imaging of exosome uptake

Freshly isolated exosomes from MDA-MB-231 cells were labeled with the PKH26 red fluorescent cell linker kit (Sigma-Aldrich, St. Louis, MO, USA) according to the manufacturer’s instructions with a small modification, and then cultured with HUVEC for 3 h. After being washed, HUVECs were stained with Hoechst for 15 min. The pictures were taken and the uptake of exosomes was observed using a fluorescence confocal microscope.

### Generation of genetically modified cells using CRISPR/Cas9 genome editing

The knockout of STIM1 in MDA-MB-231 cell lines was achieved by the CRISPR/Cas9 system. Oligonucleotide targeting exon 4 (5’-ATACAATTGGACCGTGGATG-3’) was designed as the CRISPR target site. MDA-MB-231 cells were transfected with the plasmid of the STIM1-targeted gRNA encoding SpCas9 by Lipofectamine 3000. Following 72 h of puromycin selection, the cell culture was extended for 96 h without puromycin, and the viable clonal cells were subcultured in a 96-well plate with a density of one cell/well. Afterward, cells were cultured for 7–10 days. Individual clones were expanded and screened for STIM1 depletion by genomic DNA sequencing and immunoblotting.

### miRNA isolation and real-time quantitative reverse transcription-PCR (RT-qPCR) assay

The total RNA from the cells and exosomes was isolated using the TRIzol reagent (Life Technologies, Carlsbad, CA, USA) or E.Z.N.A.^TM^ miRNA Kit (Omega Bio-Tek, Norcross, GA, USA), respectively. The cDNA synthesis was performed using M-MLV reverse transcriptase. Real-time PCR was performed using the miScript SYBR Green PCR Kit on a CFX96^TM^ Real-Time PCR Detection System (BIO-RAD, Hercules, CA, USA). The sequences of several miRNA primers are described in Table 1. The expression levels of U6 were used as an endogenous control for each sample. The relative gene expression levels were calculated using the comparative Ct (∆∆Ct) method.

**Table 1:** The sequences of several miRNA primers.

miRNA	RT-Primer	Forward	Reverse
hsa-miR-21	gtcgtatccagtgcagggtccgaggtattcgcactggatacgactcaaca	gcgcgtagcttatcagactga	agtgcagggtccgaggtatt
hsa-miR-23a	gtcgtatccagtgcagggtccgaggtattcgcactggatacgacggaaat	gcgatcacattgccaggg	agtgcagggtccgaggtatt
hsa-miR-155	gtcgtatccagtgcagggtccgaggtattcgcactggatacgacaacccc	cgcgttaatgctaatcgtgata	agtgcagggtccgaggtatt
hsa-miR-221	gtcgtatccagtgcagggtccgaggtattcgcactggatacgacgaaacc	cgcgagctacattgtctgctg	agtgcagggtccgaggtatt
hsa-miR-222	gtcgtatccagtgcagggtccgaggtattcgcactggatacgacacccag	gcgcgagctacatctggcta	agtgcagggtccgaggtatt
hsa-miR-449	gtcgtatccagtgcagggtccgaggtattcgcactggatacgacaccagc	cgcgtggcagtgtattgtta	agtgcagggtccgaggtatt
hsa-miR-494	gtcgtatccagtgcagggtccgaggtattcgcactggatacgacgaggtt	cgcgtgaaacatacacggga	agtgcagggtccgaggtatt
hsa-miR-9	gtcgtatccagtgcagggtccgaggtattcgcactggatacgactcatac	gcgcgtctttggttatctagct	agtgcagggtccgaggtatt
hsa-miR-34a	gtcgtatccagtgcagggtccgaggtattcgcactggatacgacacaacc	cgcgtggcagtgtcttagct	agtgcagggtccgaggtatt
hsa-miR-125a-3p	gtcgtatccagtgcagggtccgaggtattcgcactggatacgacagctcc	gcgacgggttaggctcttg	agtgcagggtccgaggtatt
hsa-miR-125a-5p	gtcgtatccagtgcagggtccgaggtattcgcactggatacgactcacaa	cgcgtccctgagaccctaac	agtgcagggtccgaggtatt
hsa-miR-126	gtcgtatccagtgcagggtccgaggtattcgcactggatacgaccgcatt	cgcgtcgtaccgtgagtaat	agtgcagggtccgaggtatt
hsa-miR-145	gtcgtatccagtgcagggtccgaggtattcgcactggatacgacagggat	cggtccagttttcccagga	agtgcagggtccgaggtatt
hsa-miR-146a	gtcgtatccagtgcagggtccgaggtattcgcactggatacgacaaccca	cgcgtgagaactgaattcca	agtgcagggtccgaggtatt
hsa-miR-148a	gtcgtatccagtgcagggtccgaggtattcgcactggatacgacacaaag	gcgcgtcagtgcactacagaa	agtgcagggtccgaggtatt
hsa-miR-497	gtcgtatccagtgcagggtccgaggtattcgcactggatacgacacaaac	gcgcagcagcacactgtg	agtgcagggtccgaggtatt
hsa-miR-519c	gtcgtatccagtgcagggtccgaggtattcgcactggatacgacatcctc	gcgcgaaagtgcatcttttta	agtgcagggtccgaggtatt
U6	ttcacgaatttgcgtgtcatc	cgcttcggcagcacatatac	ttcacgaatttgcgtgtcatc

### miRNA antagomir transfection

HUVECs were cultured on 6-well plates (1 × 10^5^ cells/well), and transfected with miR-145 antagomir or antagomir negative control (NC) using Lipofectamine 6000 according to the manufacturer’s instructions. Simultaneously, Exo-STIM1-KO was added. After 24 h, cells were washed twice by PBS to remove remaining exosomes or antagomir and collected to extract proteins and RNA. Chemically modified miR-145 antagomir was purchased from Shanghai GenePharma Co., Ltd.

### Matrigel plug assay for angiogenesis in nude mice

All animal experiments were conducted at Laboratory Animal Center, Institute of Radiation Medicine, Chinese Academy of Medical Sciences in accordance with the Institutional Animal Care and Use Committee guidelines. Mice were randomly assigned to four groups with three mice in each group. Briefly, 2 × 10^6^ HUVECs were mixed with Exo, Exo-STIM1-KO, miR-145 antagomir, or antagomir NC, respectively. Then Lipofectamine 6000 was added. The cell suspensions were mixed with 400 μl of High Concentration Matrigel^TM^ Matrix at a ratio of 1:4, and then the mixture was subcutaneously injected into the dorsal region of nude mice (female, 6-week-old BALB/c). After 10 days, the Matrigel plugs were harvested and processed for analysis. The degree of vascularization was evaluated by determining hemoglobin content. On the other hand, plugs were fixed with 4% formaldehyde and embedded in paraffin, and stained with hematoxylin and eosin. The vessel area was quantified as mean relative tube length by image analysis of six random microscopic fields using ImageJ software.

### Statistical analysis

All values are expressed as means ± SEM of triplicate values. One-way ANOVA followed by Tukey’s Multiple Comparison Test was utilized to determine the statistical significance with GraphPad Prism 5 (GraphPad, San Diego, CA, USA). Differences were considered statistically significant when *P* < 0.05.

## Supplementary information

Supplementary material

Supplementary figure legends

Supplementary figure 1

Supplementary figure 2

Supplementary figure 3

Supplementary figure 4
