# Supplementary material for: STIM1 promotes angiogenesis by reducing exosomal miR-145 in breast cancer MDA-MB-231 cells
Source: Cell Death Dis. 2021 Jan 4;12(1):38. doi: 10.1038/s41419-020-03304-0 (PMC7791041; doi:10.1038/s41419-020-03304-0)
Supplement: Supplementary file 1 — Supplementary material [file 41419_2020_3304_MOESM1_ESM.docx]

**STIM1 promotes angiogenesis by reducing exosomal miR-145 in breast cancer MDA-MB-231 cells**

Shunli Pan^1^, Xiaoxia Zhao^1^, Chen Shao^1^, Bingjie Fu^1^, Yingying Huang^1^, Ning Zhang^1^, Xiaojing Dou^1^, Zhe Zhang^1^, Yuling Qiu^1^, Ran Wang^1^, Meihua Jin^1^ and Dexin Kong^1,2^

^1^Tianjin Key Laboratory on Technologies Enabling Development of Clinical Therapeutics and Diagnostics, School of Pharmacy, Tianjin Medical University, Tianjin 300070, China.

^2^School of Medicine, Tianjin Tianshi College, Tianyuan University, Tianjin 301700, China.

Correspondence: Meihua Jin ([jinmeihua@tmu.edu.cn](mailto:jinmeihua@tmu.edu.cn)) or Dexin Kong ([kongdexin@tmu.edu.cn](mailto:kongdexin@tmu.edu.cn%20) )

**Materials and Methods**

**Flow cytometric analysis of cell cycle distribution and apoptosis**

The effects of A23187 and SKF96365 on cell cycle distribution and apoptosis in MDA-MB-231 cells were analyzed by flow cytometer. Briefly, MDA-MB-231 cells (2 × 10^5^ cells/ml, 2 ml) were plated in 6-well plates and treated with A23187 or SKF96365 for 24 h.The cells were collected and fixed with 70% ethanol, and stained with PI solution (25 μg/ml). The treated cells were subjected to flow cytometer FACS Verse (Becton Dickinson, Germany) for cell cycle distribution analysis.

Analysis of apoptosis was carried out by Annexin V-FITC/PI double staining. After harvested, the cells were re-suspended in 100 μl of binding buffer, incubated with Annexin V-FITC/PI solution in the dark for 15 min. Finally, samples were analyzed using flow cytometer BD Accuri C6 (BD Biosciences, San Jose, CA, USA).

Data were quantified by using Flow Jo Software (Tristar, CA, USA).

**Results**

**A23187 inhibits proliferation and induces apoptosis at high concentration, inhibits migration in MDA-MB-231 cells**

Firstly, we have tested the contamination of mycoplasma by using PCR test. The result showed that MDA-MB-231 cells were not contaminated by mycoplasma (data not shown). To investigate the *in vitro* anticancer activity of A23187, we first tested the effect of A23187 on the proliferation of MDA-MB-231 cells with MTT assay. A23187 did not effectively inhibited cell viability with concentrations below 50 nM, but showed cell cytotoxicity at concentrations higher than 100 nM in a time dependent manner (Fig. S1a). Next, we investigated the effect of A23187 on intracellular Ca^2+^ level. As shown in Fig. S1b, A23187 elevated intracellular Ca^2+^ level in a concentration dependent manner, with the intracellular Ca^2+^ level highest at 1 h after treatment. The effect of A23187 on apoptosis was determined by Annexin V-FITC/PI staining assay. As shown in Fig. S1c and S1d, increase of apoptosis was observed following treatment of A23187 at concentrations higher than 1000 nM. We also investigated the effect of A23187 on cell cycle progression, with the result of G0/G1 arrest (Fig. S1e-f). In addition, we demontrated that A23187 promoted cell migration at concentrations of 50, 100, and 200 nM, (Fig. S1g-h).

**SKF96365 suppresses proliferation and induces apoptosis in MDA-MB-231 cells**

After treating the cells for 24 h or 48 h. SKF96365 inhibited cell viability of MDA-MB-231 cells in a concentration and time dependent manner (Fig. S2a). And SKF96365 treatment increased the apoptotic cell population dose-dependently (Fig. S2b-c).

**Knockout of STIM1 by CRISPR/Cas9 in MDA-MB-231 cells**

CRISPR/Cas9 genome editing was used to edit exon 4 in the STIM1 locus (ENSG00000167323). Diagram of STIM1 gene exon 4 and gRNA targeting site were shown as Fig. S3a. We confirmed the sequence character of exon 4 in STIM1 in MDA-MB-231 cells with Sanger sequencing, showing the gene-edited MDA-MB-231 cells carrying a 1-bp deletion at the gRNA-targeting region (Fig. S3b).

**A23187 does not change miR-145 level in MDA-MB-231 cells**

MDA-MB-231 cells were treated with A23187 for 6 h, and then the level of miR-145 was measured. We found that A23187 did not alter the level of miR-145 in MDA-MB-231 cells (Fig. S4).
