# Supplementary material for: STIM1 promotes angiogenesis by reducing exosomal miR-145 in breast cancer MDA-MB-231 cells
Source: Cell Death Dis. 2021 Jan 4;12(1):38. doi: 10.1038/s41419-020-03304-0 (PMC7791041; doi:10.1038/s41419-020-03304-0)
Supplement: Supplementary file 2 — Supplementary figure legends [file 41419_2020_3304_MOESM2_ESM.docx]

**Sypplementary Figure legends**

**Fig. S1 A23187 suppresses cell viability, promotes intracellular Ca^2+^ level, induces cell apoptosis and cell cycle arrest, and promotes cell migration in MDA-MB-231 cells**

**a** Effect of A23187 on MDA-MB-231 cell viability. **b** Effect of A23187 on MDA-MB-231 intracellular Ca^2+^ level. **c** Effect of A23187 on apoptosis in MDA-MB-231 cells. **d** Quantification of the results in (c). **e** Effect of A23187 on cell cycle distribution in MDA-MB-231 cells. **f** Quantification of the results in (e). **g** Effect of A23187 on migration of MDA-MB-231 cells. **h** Quantification of the results in (g).

**Fig. S2 SKF96365 reduces cell viability, induces cell apoptosis in MDA-MB-231 cells**

**a** Effect of SKF96365 on MDA-MB-231 cell viability. **b** Effect of SKF96365 on apoptosis in MDA-MB-231 cells. **c** Quantification of the results in (b).

**Fig. S3 CRISPR/Cas9 causes targeted deletion of STIM1**

**a** Schematic diagram of STIM1 gene editing using CRISPR/Cas9 in MDA-MB-231 cells. gRNA target and PAM sequences are denoted in red and blue font, respectively. The gRNA targeting site is at the exon 4. **b** The Sanger sequencing of PCR product in STIM1-KO-MDA-MB-231 cells. The top sequence indicates the STIM1-WT sequences, with the target site underlined; and the bottom indicating the occurrence of the 1-bp deletion at the gRNA-targeting region.

**Fig. S4 Relative level of miR-145 in MDA-MG-231 cells with or without A23187 treatment**
