# Supplementary figures and images for: STIM1 promotes angiogenesis by reducing exosomal miR-145 in breast cancer MDA-MB-231 cells
Source: Cell Death Dis. 2021 Jan 4;12(1):38. doi: 10.1038/s41419-020-03304-0 (PMC7791041; doi:10.1038/s41419-020-03304-0)

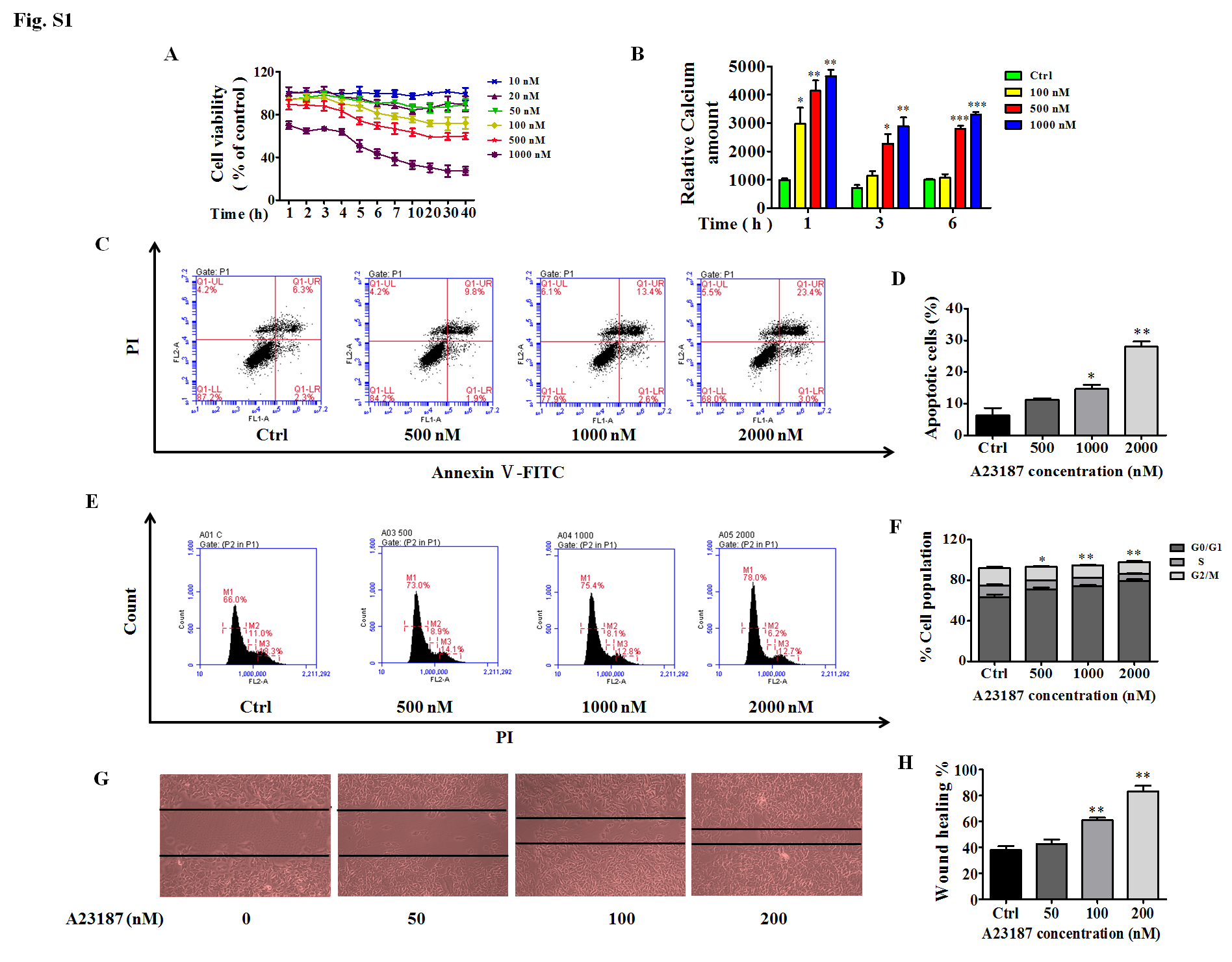

Supplement: Supplementary file 3 — Supplementary figure 1 [file 41419_2020_3304_MOESM3_ESM.tif]

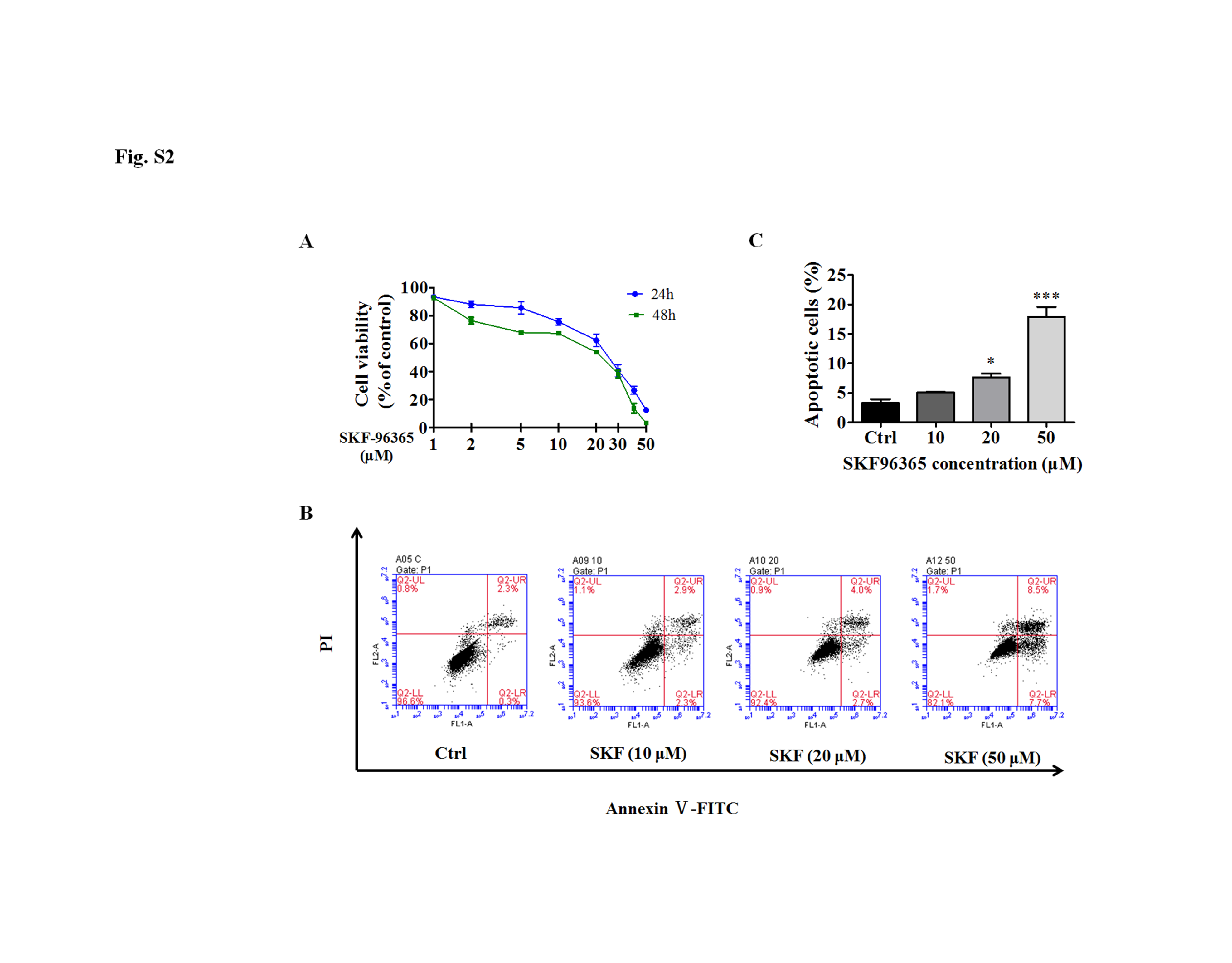

Supplement: Supplementary file 4 — Supplementary figure 2 [file 41419_2020_3304_MOESM4_ESM.tif]

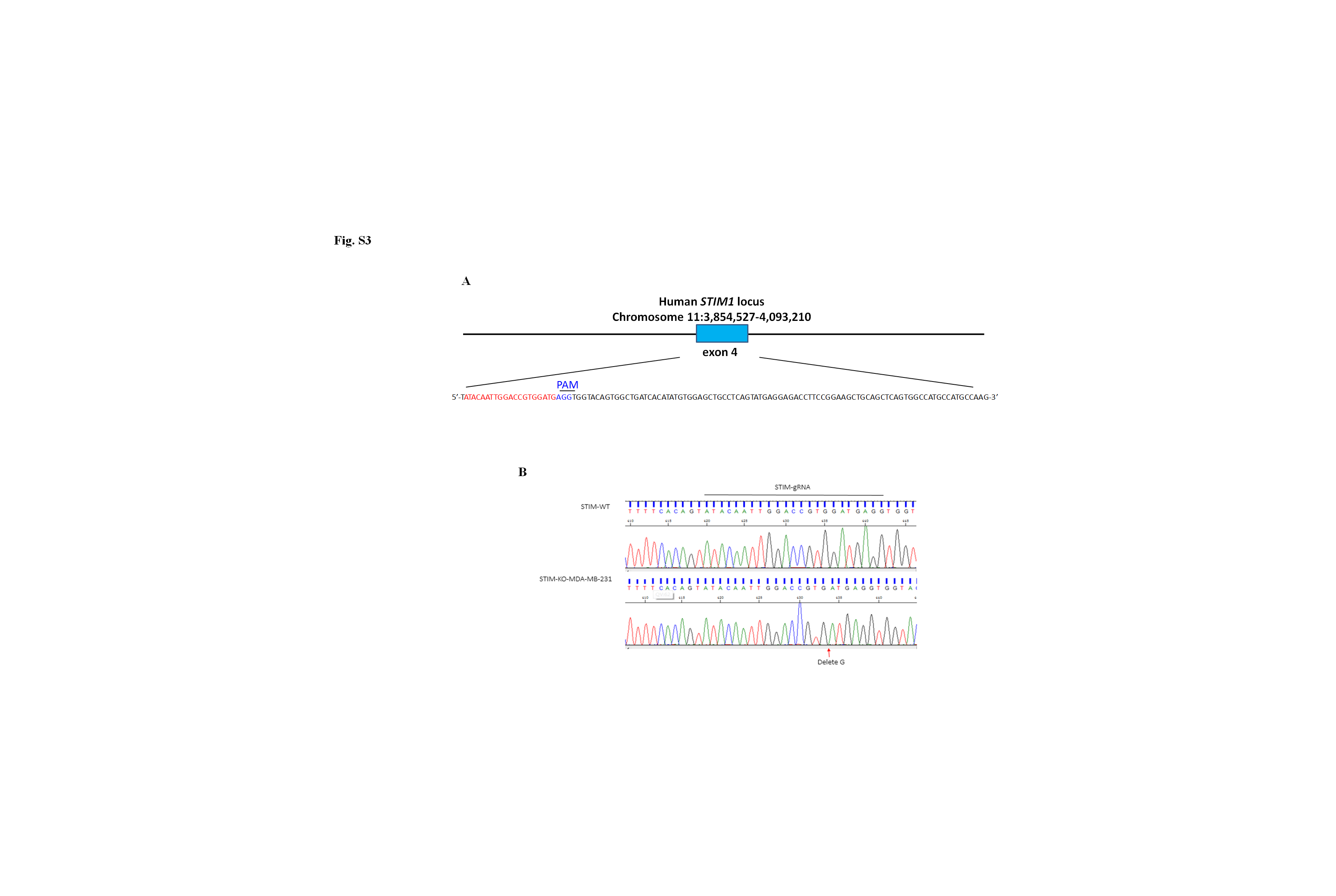

Supplement: Supplementary file 5 — Supplementary figure 3 [file 41419_2020_3304_MOESM5_ESM.tif]

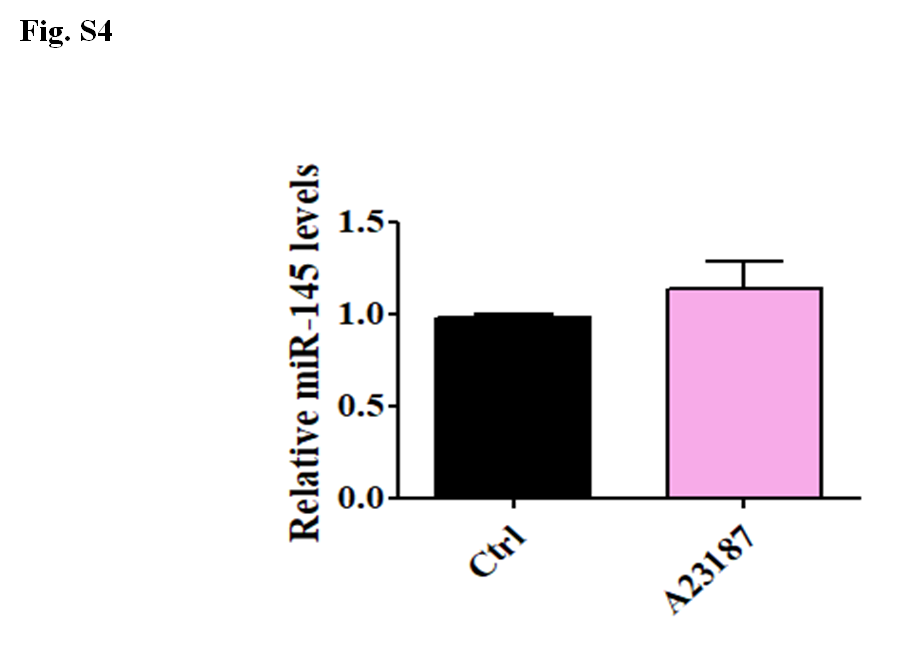

Supplement: Supplementary file 6 — Supplementary figure 4 [file 41419_2020_3304_MOESM6_ESM.tif]
